# A case with massive hemobilia long-term after internal drainage surgery for congenital biliary dilation

**DOI:** 10.1186/s40792-021-01242-3

**Published:** 2021-07-07

**Authors:** Shunryo Minezaki, Takeyuki Misawa, Makoto Watanabe, Hideki Takahashi, Takashi Koenuma, Rie Kondo, Hiroe Toyoda, Kentaro Nemoto, Hiroyuki Tsukayama, Makoto Shibuya, Keita Wada, Keiji Sano, Yasunori Ohta, Satoe Numakura, Yuko Sasajima, Hiroshi Uozaki

**Affiliations:** 1 Department of Surgery, Teikyo University School of Medicine, 2-11-1 Kaga, Itabashi-Ku, Tokyo, 173-8606 Japan; 2 Department of Pathology, Teikyo University Hospital, 2-11-1 Kaga, Itabashi-Ku, Tokyo, 173-8606 Japan

**Keywords:** Congenital biliary dilation, Hemobilia, Choledochal–gastrointestinal anastomosis (internal drainage surgery)

## Abstract

**Background:**

Currently, there is an unwavering consensus that the standard surgery for congenital biliary dilation (CBD) is extrahepatic bile duct resection and choledochojejunostomy. However, decades prior, choledochocyst–gastrointestinal anastomosis without extrahepatic bile duct resection (internal drainage surgery, IDS) was preferred for CBD because of its simplicity. Currently, there is almost no chance of a surgeon encountering a patient who has undergone old-fashioned IDS, which has been completely obsolete due to the risk of carcinogenesis from the remaining bile duct. Moreover, the pathological condition long after IDS is unclear. Herein, we report a case of life-threatening bile duct bleeding as well as carcinoma of the bile duct 62 years after IDS in a patient with CBD.

**Case presentation:**

An 82-year-old Japanese woman with hemorrhagic shock due to gastrointestinal bleeding was transferred to our hospital. She had a medical history of unspecified surgery for CBD at the age of 20. Based on imaging findings and an understanding of the historical transition of the surgical procedure for CBD, the cause of gastrointestinal bleeding was determined to be rupture of the pseudoaneurysm of the dilated bile duct that remained after IDS. Hemostasis was successfully performed by transcatheter arterial embolization (TAE) in an emergency setting. Then, elective surgery for extrahepatic bile duct resection and choledochojejunostomy was performed to prevent rebleeding. Pathological examination revealed severely and chronically inflamed mucosa of the bile duct. Additionally, cholangiocarcinoma (Tis, N0, M0, pStage 0) was incidentally revealed.

**Conclusion:**

It has been indicated that not only carcinogenesis, but also a risk of life-threatening bleeding exists due to long-lasting chronic inflammation to the remnant bile duct after IDS for CBD. Additionally, both knowledge of which CBD operation was performed, and an accurate clinical history are important for the diagnosis of hemobilia.

## Background

Hemobilia is caused by iatrogenic factors, trauma, and neoplasms, and other causes are rare [1]. Although there have been few case reports, hemobilia can be caused by chronic inflammation or arteriovenous malformation [2, 3]. The most common cause of hemobilia due to chronic inflammation is bile duct stones. Therefore, there are very few reports of congenital biliary dilation (CBD)-related hemobilia.

Currently, there is an unwavering consensus that the standard surgery for CBD is extrahepatic bile duct resection and choledochojejunostomy [4]. However, decades prior, choledochocyst–gastrointestinal anastomosis without extrahepatic bile duct resection (internal drainage surgery, IDS) was the mainstream procedure for CBD because of its simplicity and the lack of knowledge about carcinogenesis from the remnant bile duct [5]. However, even today, we surgeons may sometimes encounter the patient who has undergone IDS that has been obsolete due to the risk of carcinogenesis. Moreover, little is known about the pathological condition long after IDS other than carcinogenesis in CBD. Herein, we report a case of bile duct bleeding as well as carcinoma in situ (CIS) of the bile duct 62 years after IDS for CBD.

## Case presentation

An 82-year-old Japanese woman presented with hemorrhagic shock. In the primary hospital, she was diagnosed with a hemorrhagic pancreatic pseudocyst on computed tomography (CT), and then transcatheter arterial embolization (TAE) was performed. However, hemostasis was not obtained, and the patient was transferred to our hospital for close examination and treatment. Laboratory examination showed a red blood cell count of 401 * 10^4^/µl, a platelet count of 39.4 * 10^4^/µl, a white blood cell count of 13,300/µl, an aspartate aminotransferase level of 806 U/L, an alanine aminotransferase level of 443 U/L, a lactate dehydrogenase level of 736 U/L, an alkaline phosphatase level of 2664 U/L, a γ-glutamyl transpeptidase level of 545 U/L, a pancreatic-type amylase level of 66 U/L, a C-reactive protein level of 1.35 mg/dl, a carcinoembryonic antigen (CEA) level of 2.2 ng/ml, and a carbohydrate antigen 19-9 (CA19-9) level of 217.5 U/ml. Mild anemia, inflammation, and elevated CA19-9 and pancreatic-type amylase levels were revealed. The patient had a history of surgery for CBD in the 1950s. However, the details of the surgical procedure are unknown. There was no history of pancreatic disease or drinking.

**Fig. 1:**
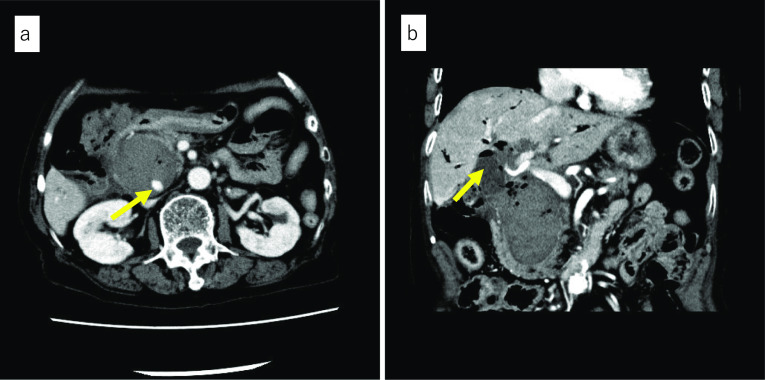
Preoperative enhanced CT findings. **a** Transverse image. Contents of the cystic lesion of the pancreatic head reveal a relatively high CT number. An arrow shows pseudoaneurysm in the cystic lesion. **b** Coronal image. The gallbladder had been resected. An arrow shows the suspicious structure of the elevated jejunum adjacent to the gallbladder bed

**Fig. 2:**
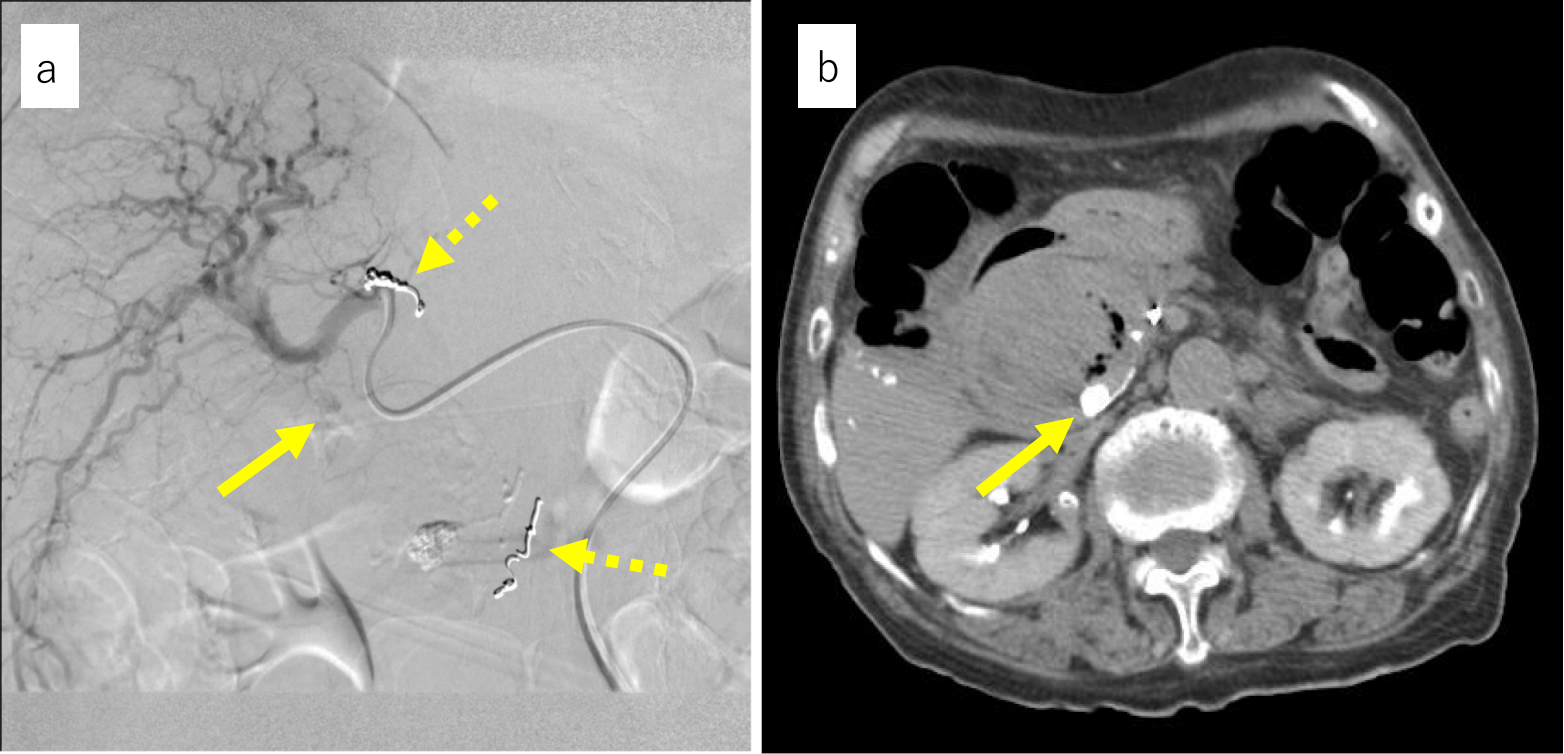
Findings from angiography performed in our hospital. **a** Contrast image of the proper hepatic artery is shown. The inferior pancreaticoduodenal artery and branch of the proper hepatic artery were coiled at a previous hospital (dotted arrow). A solid arrow shows extravasation. The culprit vessel was suspected to be the PBP, which is a branch of the proper hepatic artery. **b** Transverse CT image after embolization with lipiodol and n-butyl-2-cyanoacrylate is shown. An arrow shows embolized pseudoaneurysm

Enhanced CT at our hospital showed high-density collection, which indicated rather flesh blood and pseudoaneurysm within the cystic lesion measuring 50 mm in the pancreatic head (Fig. 1a). The gallbladder had already been resected, and the intestinal tract, which was suspected to be the lifted jejunum, was adjacent to the cystic lesion (Fig. 1b). Angiography was reperformed for diagnosis and treatment at our hospital on the day of admission, and extravasation from the pseudoaneurysm measuring 11 mm in the peribiliary vascular plexus (PBP) branched from the proper hepatic artery was recognized. The PBP was embolized as selectively as possible with n-butyl-2-cyanoacrylate (NBCA), and hemostasis was obtained (Fig. 2a, b). The clinical course after TAE was uneventful and no liver dysfunction occurred. Based on the CT findings, interventional radiology (IVR), and considerations that IDS was the standard operation for CBD in the 1950s, the cause of hemorrhage shock was determined to be due to hemobilia of the remaining dilated bile duct after IDS.

**Fig. 3:**
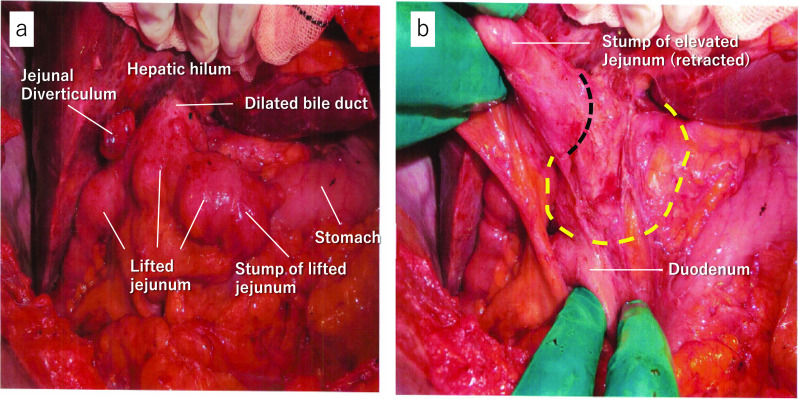
Intraoperative photographs which prove that the previous surgery was IDS. **a** Lifted jejunum is anastomosed to the ventral side of dilated bile duct with side-to-side anastomosis. A jejunal diverticulum was seen (cholecystectomy had been performed with this patient). **b** Dilated bile duct and anastomosis are exposed after mobilization of the lifted jejunum. A yellow dotted line shows dilated bile duct. A black dotted line shows side-to-side anastomosis with lifted jejunum (IDS)

After the general condition of the patient improved, resection of the extrahepatic bile duct with bilioenteric reanastomosis was performed to prevent rehemorrhage 16 days after TAE. The operation performed 62 years ago was determined as IDS in which side-to-side anastomosis was made with the lifted jejunum and dilated bile duct in a Roux-en-Y fashion (Fig. 3a, b). The resected extrahepatic bile duct presented severe adhesions due to chronic inflammation. During dissection around the dilated bile duct, special attention was paid not to damage the right hepatic artery by checking its pulsation with palpation and ultrasonic probe. Since the inflammation around the dilated bile duct was severe, the dissection between the bile duct wall and the pancreas head was troublesome. For fear of insufficient closure of the biliary stump, complete dissection of the bile duct was abandoned, and the anal stump of the common bile duct was closed slightly caudal to the hemorrhagic point which was identified by the clot on the inner lumen of the dilated bile duct. Reanastomosis with the oral stump of the bile duct and the elevated jejunum used for the prior surgery was performed. Although a postoperative pancreatic fistula (Clavien–Dindo classification II) developed, the patient had an almost uneventful postoperative course. Postoperative recovery was relatively slow due to the patient's advanced age. The patient was transferred to a rehabilitation hospital 28 days after surgery, including a 1-week waiting period. After follow-up for 2 years, there were no symptoms of rehemorrhage or cholangitis.

**Fig. 4:**
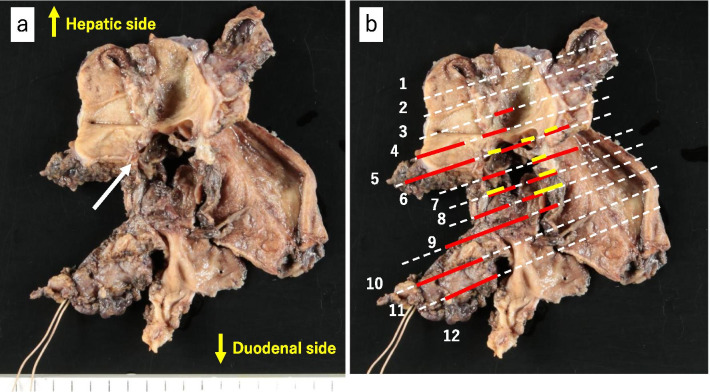
Macroscopic findings of the resected specimen of extrahepatic bile duct. **a** Suspected bleeding site with blood clot, severe inflammation, and existence of embolic material (solid white arrow). **b** A white dotted line with serial number is secant line for pathological examinations. Red lines indicate hemorrhagic area, and a yellow line indicates carcinoma area

**Fig. 5:**
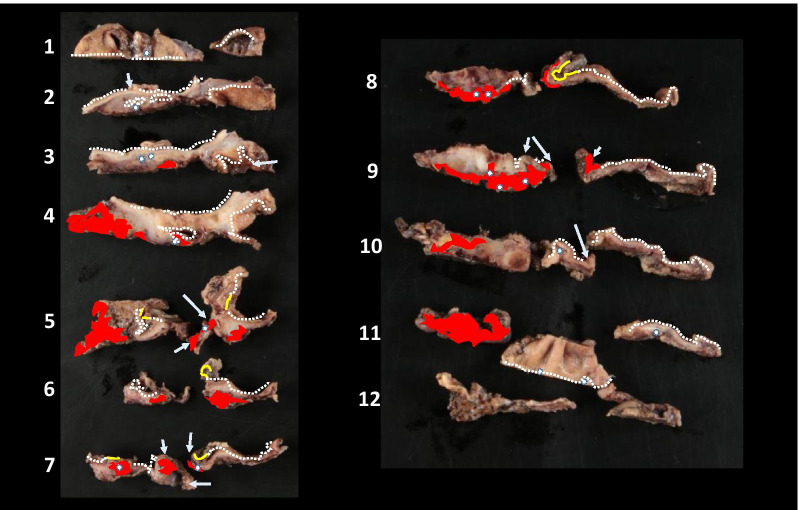
Sequential sections of resected specimen with serial number. White dotted lines indicate non-tumoral epithelium. The CIS (yellow lines) and the hemorrhage sites (red areas) including the ulceration sites (white arrow) are distant. Embolic material (white spot) is observed in the hemorrhage site, and an ulcer and superficial vascular aggregates are observed at the sites irrelevant to the CIS

**Fig. 6:**
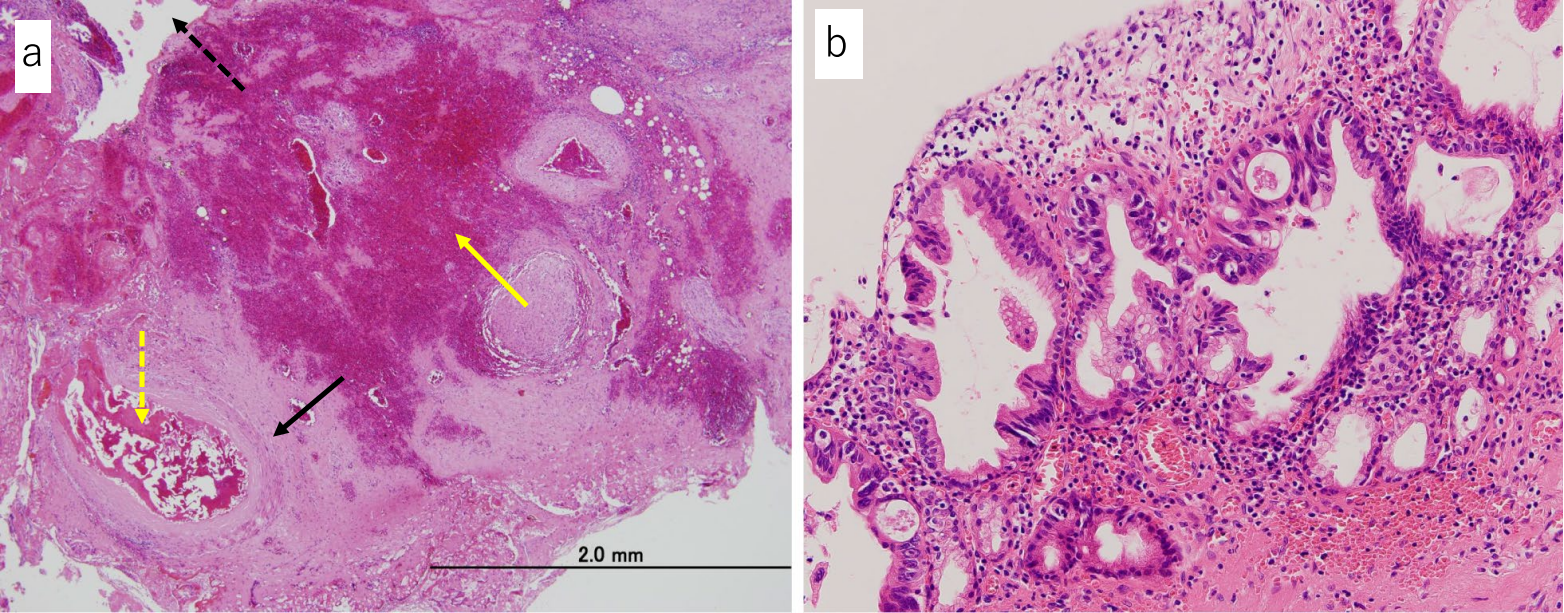
Histological findings following hematoxylin and eosin staining. **a** Hemorrhage under the bile duct epithelium (dotted black arrow) at a site irrelevant to the CIS. Embolic material (dotted yellow arrow) is observed in the ruptured artery (solid black arrow), which is interpreted as the cause of hemorrhage. Hemorrhage site (solid yellow arrow) is accompanied by vascular proliferation that is considered to have existed before the hemorrhage. **b** Image of the CIS. No ulcer was observed

Resected specimen of extrahepatic bile duct (Fig. 4a, b) was severely inflamed, and was processed into sequential sections (Fig. 5) for the pathological examinations. Carcinoma and scattering ulcerative lesions in the bile duct mucosa were microscopically and pathologically diagnosed on these sections. Obstruction with embolic material used in TAE was observed in the ruptured artery, and hemorrhage was revealed in the bile duct lumen. The hemorrhage site was accompanied by vascular proliferation and severe inflammation that were considered to have existed before the hemorrhage (Fig. 6a). As for the ruptured artery, the loss of elastic fibers was recognized and suggested that an aneurysm might have been present. Additionally, incidental CIS was found at a distance from hemorrhage site on the membrane of the bile duct. The patient was diagnosed with well-differentiated adenocarcinoma, TisN0M0, pStage0, R0 (Fig. 6b).

## Discussion

Hemobilia is caused by iatrogenic factors, trauma, and neoplasms. In recent years, hemobilia has mainly been caused by iatrogenic factors, including IVR, which can be explained by the popularization of hepatopancreatobiliary intervention [6]. Hemobilia is a rare symptom, even more rare, except for traumatic and iatrogenic causes. Therefore, the diagnosis of hemobilia is generally difficult. Because the nothing of unique findings of hemobilia, not only various diagnostic imaging techniques, but also a detailed clinical history is important for its diagnosis [7]. Additionally, the diagnosis of hemobilia was difficult in this case, as a previous doctor diagnosed the cause of hemorrhage as a hemorrhagic pancreatic pseudocyst. Records of the operation performed for CBD 62 years prior were not available. Based on the CT findings, IVR images, and considering the standard operative method for CBD in the 1950s, we determined that the patient underwent IDS for CBD instead of division surgery which is currently used as a standard operation.

The purposes of treatment for hemobilia are hemostasis and maintaining bile flow. The latter is especially important because of the development of cholangitis or pancreatitis due to blood clots in the biliary tract [8]. Various treatments for hemobilia, including follow-up observation, endoscopic approaches, and surgery, depend on the cause or degree of hemorrhage. IVR, as a diagnostic and treatment modality, is effective for patients for whom hemodynamics are unstable due to active hemorrhage [7]. In our case, IVR was chosen because the patient was in hemorrhagic shock. Generally, the blood flow of the extrahepatic bile duct is supplied from the left and right hepatic arteries, cystic artery, proper hepatic artery, and posterior superior pancreaticoduodenal artery. These arteries form the complex PBP [9]. Additionally, considering that extravasation was not observed in the supplying arteries to the pancreatic head from the pancreaticoduodenal arcade, the patient was diagnosed not with hemorrhage from pseudopancreatic cysts but with hemorrhage in the remnant dilated bile duct lumen. Furthermore, in our case, extravasation occurred from a pseudoaneurysm developed in the branch of the proper hepatic artery. Although selective TAE was successfully performed and hemostasis was obtained, there was concern about the possibility of rehemorrhage from the PBP. Therefore, extrahepatic bile duct resection with bilioenteric reanastomosis was performed to prevent rehemorrhage.

For a long time, CBD had been considered an indication for surgery because it was known to cause cholangitis, obstructive jaundice, hepatitis, and spontaneous rupture, and many untreated patients died after follow-up [10]. However, division surgery (i.e., resection and reconstruction of the extrahepatic bile duct to prevent oncogenesis) was not generally performed. Therefore, the standard operation for CBD was simple IDS (i.e., anastomosis between the gallbladder and nearby digestive tract or between the extrahepatic bile duct and digestive tract). However, gallbladder–neighbor digestive tract anastomosis was problematic, as bile excretion was not satisfactory. On the other hand, extrahepatic bile duct resection was rarely performed since the procedure is complicated and thus leads to high mortality. Therefore, IDS in which the dilated bile duct and digestive tract were directly anastomosed (e.g., choledochocystoduodenostomy or choledochocystojejunostomy in a Roux-en-Y fashion) was usually chosen as the standard operation to prevent reflux cholangitis and secure an improvement of jaundice [10]. Later, the risk of oncogenesis after IDS was reported [11]. Because IDS has an approximately fourfold increased risk of oncogenesis compared with extrahepatic bile duct resection and gallbladder resection with bile tract reconstruction, IDS is not currently the standard operation [12, 13]. In fact, approximately 21.6% of adult CBD patients with biliary dilation were revealed to develop cholangiocarcinoma during the natural course [14]. According to the Cancer Registry and Statistics, Cancer Information Service, National Cancer Center, Japan (Vital Statistics of Japan), the incidence rate of cholangiocarcinoma in Japanese patients is 0.018% [15]. Therefore, CBD patients with biliary dilation have an approximately 1200 times higher risk of cholangiocarcinoma. In our case, CIS was found incidentally in a very limited location during the pathological examination. Furthermore, regardless of the location of the CIS, abnormal angiogenesis and microscopic hemorrhage were extensively observed under the mucosal epithelium of the extracted bile duct. Based on these findings, CIS and bleeding are unrelated, and the formation and collapse of pseudoaneurysm due to chronic inflammation over many years was considered the cause of biliary hemorrhage.

In the case of cholelithiasis, it is generally known that chronic inflammation causes erosion or ulceration in the gallbladder mucosa, followed by rupture of the submucosal artery, and pseudoaneurysm is formed due to repeated hemorrhage and thrombolization [16]. In the case of CBD, bile and refluxed pancreatic juice are mixed in the bile duct due to the pancreaticobiliary maljunction, activating pancreatic juice with bacterial infection, and continuous inflammation is present in the bile duct mucosa [17]. Therefore, the cause of hemobilia was interpreted to be pseudoaneurysm that developed and ruptured due to long-term and severe chronic inflammation of the bile duct mucosa [18]. In our case, to pathologically identify the ruptured pseudoaneurysm, we performed EVG staining which highlights elastic fibers of blood vessels. However, we could not find any responsible lesions probably because the bile duct was so inflamed that we resected the specimen piece by piece. Therefore, we diagnosed that hemobilia was due to rupture of pseudoaneurysm based on IVR findings and clinical features. IVR showed a clear pseudoaneurysm, and the clinical course showed that the patient had been in shock status due to massive gastrointestinal bleeding and quickly recovered after embolization of the pseudoaneurysm. Additionally, it was interpreted that the bile duct mucosa was exposed to severe chronic inflammation for a long time because an activated pancreatic juice, intestinal juice or bacteria flowed into the remnant dilated bile duct through the anastomosis of IDS.

Generally, massive hemorrhage is rare in hemobilia because hemorrhage is suppressed with high intraluminal pressure, especially in the case of a thickened bile duct due to chronic inflammation [19]. Although a thickened bile duct, which is a characteristic of CBD, was observed in our case, the most notable point is that the intraductal pressure could not increase to suppress bleeding because the blood flowed out into the jejunum through the anastomosis [20]. Thus, the patient was believed to have easily developed hemorrhagic shock associated with massive hemobilia.

A search of PubMed using the keywords "haemobilia", "hemobilia", "choledochal cyst", and "congenital biliary dilation" from 1948 to 2020 revealed only two cases of hemobilia in CBD. One report reviewed hemobilia from a ruptured pseudoaneurysm due to inflammation in a 21-month-old girl. Hemostasis was obtained with TAE, and division surgery was performed [21]. The other report reviewed hemobilia from benign biliary papillomatosis in a 57-year-old woman. Division surgery was performed, and the patient was followed up uneventfully for 4 years after surgery [22]. Our case which exhibited hemobilia in the CBD patient had been performed IDS is the first report in the world literature. 

## Conclusion

We report a case of massive hemobilia in a patient with CBD who underwent IDS for CBD 62 years ago. At present, a certain number of patients who have undergone IDS for CBD remain alive. When encountering hemobilia in the clinic, it is important to have an accurate medical history of the potential for CBD and any related surgical procedures.
